# A randomised controlled trial evaluating family mediated exercise (FAME) therapy following stroke

**DOI:** 10.1186/1471-2377-8-22

**Published:** 2008-06-20

**Authors:** Rose Galvin, Tara Cusack, Emma Stokes

**Affiliations:** 1 Department of Physiotherapy, School of Medicine, Trinity College Dublin, Ireland; 2 School of Physiotherapy and Performance Science, College of Life Sciences, University College Dublin, Ireland

## Abstract

**Background:**

Stroke is a leading cause of disability among adults worldwide. Evidence suggests that increased duration of exercise therapy following stroke has a positive impact on functional outcome following stroke. The main objective of this randomised controlled trial is to evaluate the impact of additional family assisted exercise therapy in people with acute stroke.

**Methods/Design:**

A prospective multi-centre single blind randomised controlled trial will be conducted. Forty patients with acute stroke will be randomised into either an experimental or control group. The experimental group will receive routine therapy and additional lower limb exercise therapy in the form of family assisted exercises. The control group will receive routine therapy with no additional formal input from their family members. Participants will be assessed at baseline, post intervention and followed up at three months using a series of standardised outcome measures. A secondary aim of the project is to evaluate the impact of the family mediated exercise programme on the person with stroke and the individual(s) assisting in the delivery of exercises using a qualitative methodology. The study has gained ethical approval from the Research Ethics Committees of each of the clinical sites involved in the study.

**Discussion:**

This study will evaluate a structured programme of exercises that can be delivered to people with stroke by their 'family members/friends'. Given that the progressive increase in the population of older people is likely to lead to an increased prevalence of stroke in the future, it is important to reduce the burden of this illness on the individual, the family and society. Family mediated exercises can maximise the carry over outside formal physiotherapy sessions, giving patients the opportunity for informal practice.

**Trial Registration:**

The protocol for this study is registered with the US NIH Clinical trials registry (NCT00666744)

## Background

Stroke is a leading cause of disability among adults in developed countries [1]. The progressive increase in the population of older people is likely to lead to an increased prevalence of stroke in the future [2]. In contrast to coronary heart disease and cancer, the burden of stroke lies with long-term disability as opposed to death. Any rehabilitation intervention that can speed recovery and reduce long-term disability would have a major impact on both the individual and the social burden of this illness. One major component of rehabilitation after a stroke is exercise therapy which serves to minimise the effects of the brain cell damage and optimise re-learning [3-5]. In this manuscript, we describe a novel protocol to increase exercise therapy time following stroke by involving the family members in the delivery of additional exercises to people with stroke.

Physiotherapists have traditionally been the mediators of exercise therapy post-stroke. Over the years different physiotherapy approaches have been advocated to promote motor recovery, including the methods of Bobath, Brunnstrom, Rood and the Proprioceptive Neuromuscular Facilitation (PNF) technique [6,7]. All of these therapy approaches are exercise based and there is evidence that physiotherapy and occupational therapy, using a mix of components from these different approaches, is significantly more effective than no treatment or placebo control in the recovery of functional independence following stroke [6]. Nonetheless, it has been suggested that the duration of exercise therapy that is delivered post stroke is, at best, 'homeopathic' [8].

Evidence from two systematic reviews [9,10] has suggested that a more intensive exercise therapy input is associated with enhanced improvement of the performance of functional activities after stroke, although the exact dose of practice required for significant functional improvements to take place is lacking. A later meta-analysis [11] also demonstrated that additional exercise therapy has a positive outcome on gait speed and activities of daily living. However this meta-analysis included studies where no formal exercise therapy was documented in the 'control' group [12-15]. The authors completed a systematic review and meta-analysis [17] that examined the effects of additional exercise therapy time by comparing randomised controlled trials that provided 'routine' therapy to the control group versus studies that provided 'routine' therapy together with 'additional' exercise therapy to the intervention group. The findings demonstrated that increased duration of exercise therapy time had a small but positive effect on activities of daily living as measured by the Barthel Index. Although the meta-analysis of the lower extremity outcome measures lacked significant findings, very few studies were included in the analysis and the results were supportive of the hypothesis that more exercise therapy improved gait speed and lower limb impairment in people with stroke. The review also highlighted the need for further randomised controlled trials with large sample sizes to evaluate the effectiveness of increased duration of exercise therapy on lower extremity outcome [17].

While the available evidence suggests that more physiotherapy is better, additional therapy is expensive and not routinely funded routinely by the National Health Service in the UK or by health insurance companies [18]. Therefore, research is now focusing on novel ways of increasing the duration of exercise therapy that occurs following stroke with minimal use of resources [8]. One suggestion has been that 'physiotherapists need to develop strategies whereby patients and caregivers take full responsibility for the bulk of therapy – for instance, training of balance, strength and endurance, repetition of simple tasks, group therapy, fitness-related training and family involvement' [19]. To date no randomised controlled trial (RCT) has evaluated the delivery of exercise by people who are not health care workers, although in a RCT by Lincoln, Parry and Vass [20], both qualified physiotherapists and physiotherapy assistants delivered two different forms of additional exercise therapy and no differences were noted between the groups. The following paper reports on the design of an evidence-based, user informed and centred RCT to evaluate the impact of increased duration of family mediated exercise (FAME) therapy in people with stroke.

### Background Work

A full systematic review and meta-analysis of previous work in this area was completed and has been accepted for publication [17]. The meta-analysis supported the theory that additional exercise therapy has a positive impact on outcome following stroke. However, the systematic review highlighted issues with patient compliance, particular patient subgroups, type of therapy, number of additional minutes of exercise therapy delivered to patients and long terms benefits of additional exercise therapy. These issues were considered in the development of the FAME RCT.

In addition, three user surveys were conducted to explore the views of potential participants in the FAME programme i.e. the individual with stroke, 'family members/friends' of people with stroke and the physiotherapists. A self-report questionnaire was administered to 100 'family members/friends' and 75 people with stroke. Two focus groups were conducted with ten expert physiotherapists working in the area of stroke rehabilitation. The results of the three user surveys supported and informed the development of the FAME trial. Family members of people with stroke indicated that they were willing to participate in the delivery of unsupervised exercises in the hospital and the home setting (n = 91). Furthermore, this method was also acceptable to people with stroke (n = 65) as an adjunct to routine physiotherapy. Physiotherapists highlighted a number of factors that influenced participation in physical therapy such level of interest and motivation of the family (n = 5), availability (n = 3) and importance of education (n = 2). 'Family members/friends' also identified reasons that would also limit participation such as work commitments (n = 24), lack of confidence (n = 20) and unsuitable treatment times (n = 13). The results of these user surveys are described elsewhere [21-24].

### Aims and Objectives of the Study

The aim of the study is to evaluate the functional recovery in two groups of primary stroke patients presenting with moderate/severe disability over a six month period through the implementation of a randomised controlled trial. The first group or the experimental group will receive routine therapy and additional lower limb exercise therapy in the form of family assisted exercises. The second group or the control group will receive routine therapy with no additional formal input from their family members. A secondary aim of the project is to evaluate the impact of the FAME programme on the person with stroke and the individual(s) assisting in the delivery of exercises.

### Ethical Considerations

The study has obtained ethical approval from four clinical sites: Saint Vincent's University Hospital Ethics and Medical Research Committee (Ref 9/5/07), Saint Colmcilles Hospital (Ref 9/5/07), the Mater Misercordiae University Hospital Research Ethics Committee (Ref 1/378/1138) and Beaumont Hospital Ethics (Medical Research) Committee (Ref 08/19).

## Methods

### Study Design

A prospective single-blind randomised controlled trial will be conducted. Forty subjects will be randomised into either an experimental group or a control group using sealed, computer generated random numbers. The progression from screening and enrollment to randomisation is illustrated in Figure legend 1.

**Figure 1:**
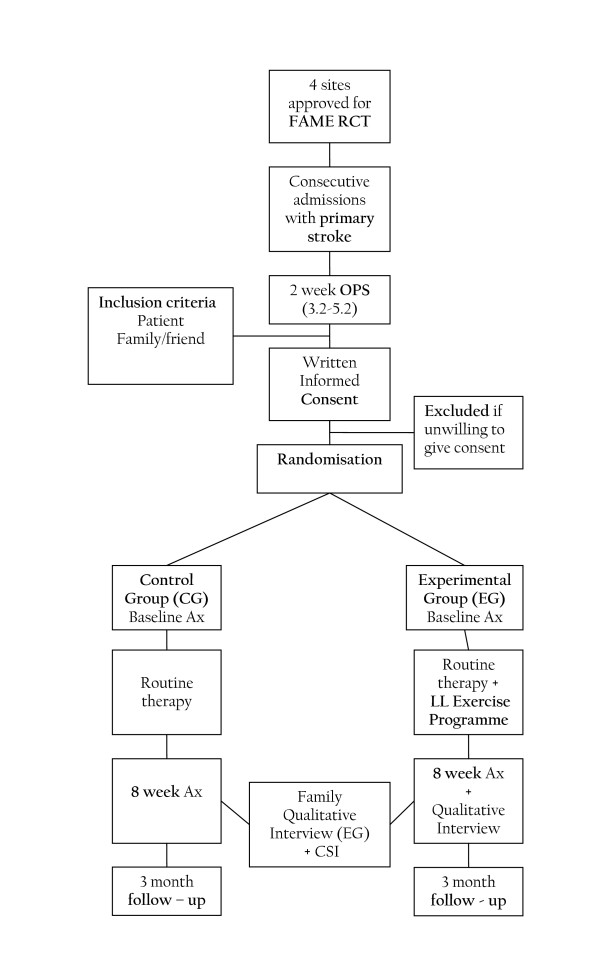
Outline of FAME study design.

### Intervention

Members of the control group and the experimental group will receive 'routine' physiotherapy for the duration of the trial, which will be delivered by the physiotherapy staff in the hospital. In addition, participants in the experimental group will be given individualised FAME programmes to be conducted at the bedside with his/her nominated 'family member/friend'. Each programme will consist of training the 'family member/friend' with the skills necessary to carry out the exercise-training programme with the person with stroke on a weekly basis. This specialised intervention will be designed and delivered by the research physiotherapist (RG). Exercises will be designed appropriate to the participants' ability. The emphasis of the lower limb exercise intervention will be on achieving stability and improving gait velocity and lower limb strength, based on patterns derived from findings reported in a systematic review of 151 intervention studies on stroke rehabilitation [25].

Based on the output from the systematic reviews and meta-analyses [11,17], it was determined that the trial would continue for eight weeks with an expectation that at least 1200 additional minutes of family mediated exercise therapy will be delivered over this time period. Each FAME session is expected to last 35 minutes and will be conducted on a daily basis. The nominated 'family member/friend' will be requested to fill out a daily exercise diary following completion of the prescribed exercises to document compliance.

### Blinding

An independent physiotherapist blinded to group allocation will assess all participants using the battery of outcome assessments on entry to the study, on completion of the eight-week trial and finally at the three month follow-up stage.

### Outcome

The primary outcome measure used in this trial will be the lower extremity section of Fugl Meyer (FM) Assessment [26]. The FM is considered by many in the area of stroke rehabilitation to be one of the most inclusive quantitative measures of sensorimotor impairment following stroke and its use has gained international acceptance as a feasible and appropriate clinical and research tool for evaluating changes in motor impairment following stroke [27]. Excellent intra-rater and inter-rater reliability and construct validity have been demonstrated [28,29].

A series of secondary outcome measures will also be used to evaluate participants' recovery including the Motor Assessment Scale [30], the Berg Balance Scale [31], the Six Minute Walk Test [32] the Barthel Index [33], the Reintegration to Normal Living Index [34] and the Nottingham Extended Activities of Daily Living Index [35].

The Caregiver Strain Index [36], a 13-item self-report measure designed to measure strain relating to care provision, will be administered to the nominated 'family member/friend' of the person with stroke. In addition, a semi-structured interview will take place with each participant and their nominated 'family member/friend' individually, in relation to their experience of the exercise therapy programme. A physiotherapist unknown to the participant will conduct interviews. All interviews will be audio recorded.

### Participant Selection

Potential participants will be recruited from four acute hospitals. A register of all stroke patients admitted to these hospitals will be sent to the research physiotherapist on a weekly basis. Those patients who are survivors at two weeks following the onset of stroke will be assessed using the Orpington Prognostic Scale. The Orpington Prognostic Scale was developed by Kalra and Crome [37] in order to stratify stroke patients according to severity of stroke. The Orpington Prognostic Score (OPS) is a clinically derived score that incorporates measures of cognitive impairment, motor deficit, balance and proprioception. The score ranges from 1.6 (best prognosis/lowest level of disability) to 6.8 (worst prognosis/highest level of disability). It has its highest predictive power of levels of dependence when assessed at two weeks following the onset of stroke [37-39]. Only patients who achieve a score from 3.2 – 5.2 on the OPS at two weeks post stroke will be recruited to this study. This cohort of patients has been described as the *'middle group' *of stroke patients and consists of people presenting with a moderate/severe deficit following stroke [38]. It has been suggested that outcome in this band of patients depends on extrinsic factors such as the intensity and quality of rehabilitation, family support, the personality and motivation of the patient and the availability of statutory and voluntary support systems in the community [38]. The scale has been validated for both an elderly and an Irish stroke population [37,40]. The test-retest and inter-rater reliability of the OPS has also been established [40].

### Inclusion/Exclusion Criteria

Patients will be admitted to the study if they present with a formal diagnosis of first unilateral stroke, are over 18 years, are willing to give informed consent and have 'family members/friends' willing to participate in their assigned physiotherapy intervention programme. Patients will be excluded if they present with hemiplegia of a non-vascular origin, are discharged from hospital at baseline less than two weeks following stroke, have an OPS score of less than 3.2 or greater than 5.2 at two weeks following stroke onset, have a pre-existing neurological disorder resulting in a motor deficit in addition to that resulting from the stroke, present with any lower extremity orthopaedic condition such as recent fractured femur or amputation or have receptive/expressive dysphasia.

'Family members/friends' will be included if they have been nominated by the person with stroke to assist him/her in the performance of their prescribed exercises and are medically stable and physically able to assist in the delivery of exercises to the person with stroke. Suitability will be determined in liaison with the physiotherapist in charge on the patients' routine care. Family members/friends' will also be required to give informed written consent agreeing to help their 'family member/friend' with stroke in the performance of his/her prescribed exercises.

### Recruitment

The research physiotherapist (RG) will be responsible for assessing eligibility for inclusion into the study and also for obtaining informed consent from the patient and his/her 'family member/friend'. Following identification of suitable participants i.e. people with stroke and their nominated family member/friend, the aims of the project, including the role of the participant and his/her "family member/friend" in the FAME programme, will be outlined and any questions answered by RG. The person with the stroke and his/her 'family member/friend' will each receive a separate information brochure in advance of being asked to give written informed consent agreeing to participate. Participants will have seven days between receipt of the information brochure and being requested to give written permission. Following the 7-day interval, RG will answer or clarify any further questions that arise and both the person with stroke and his/her 'family member/friend' will be requested to sign a consent form in the presence of each other. If either the patient or his/her 'family member/friend' are unwilling to give written consent, they will be excluded from the study. If they decide to partake in the study, participants will be advised that they can withdraw from the study at any time and that the study will not interfere with their routine rehabilitation programme. Participants will be allocated a reference code. Names and other details that may identify the participants will be removed.

### Randomisation

To minimise the possibility of recruitment bias, a person independent of the recruitment process will complete random group allocation. Computer generated random numbers will be kept in pre-sealed envelopes in a locked drawer. Allocation will be revealed after recruitment by a telephone call from RG to an independent person, who will open the next envelope in the sequence and give the randomisation information to RG. Each envelope will only be opened on enrollment of an eligible participant. After allocation has been revealed, the appropriate intervention will be organised by RG.

### Power

Sample size is based on a power analysis. A sample size calculation was performed for the primary outcome variable, which is the lower extremity section of the Fugl-Meyer (FM). For the FM lower extremity, a change of greater than five points reflects a change greater than measurement error [42]. Power calculations indicate that a total of 40 participants are needed in order to detect with 80% power a difference of 20% between the groups at a significance level of 5%. It is anticipated that it will take 18–24 months to recruit the required number of participants.

### Analysis

All data will be collected on paper and the records will be stored by registration number in a secure cabinet. Anonymised data will be transferred to a computer database and secured using a password. An independent researcher will cross check all entries. Appropriate statistical tests will be carried out on the data using MINITAB Release 13.1.

The statistical analyses will be performed according to the 'intention to treat' principle and participants will be analysed in the group to which they were randomly assigned. Participants who withdraw from the experimental group but allow further data collection will have data collected. A 'last measurement carried forward' method is used to predict outcome in dropouts [43].

The purpose of the statistical analysis is to test the hypothesis that there will be a clinically significant difference in functional recovery between participants in the control group and the experimental group from baseline to post-intervention assessment. Firstly, the baseline scores of the participants' demographic, primary and secondary outcomes will be compared in the two groups. If necessary, adjustments for baseline variables will be made using analysis of covariance. Analyses will be carried out to examine the difference between the groups with respect to the change in the lower extremity FM test score from baseline to post-intervention assessment. Secondary outcomes will be compared between the two groups using Student's t-test or the Wilcoxon rank sum test for ordinal data. Descriptive statistics will also be used to represent demographic data.

All interviews will be tape recorded, transcribed and examined in terms of themes according to the method devised by Miles and Hubberman [44]. The responses from all participants to each question will be transferred to Microsoft Excel for examination. Following an examination of the responses to each question, a coding system will be developed in order to facilitate the identification of recurrent responses. Three researchers will be provided with the responses to all of the questions in an unencoded format; thereafter they will independently code the responses sequentially using the predefined codes. All coding disagreements will be resolved through discussion.

## Discussion

The concept of family mediated exercise therapy is not a new phenomenon. It is common practice in rehabilitation of children with neurological impairments [45]. While it cannot be denied that the sudden and profound effects of stroke on family roles and functioning create stress for the stroke survivor and their families, the available evidence suggests that carers and families want information and want to be more fully involved in their rehabilitation [46,47]. It is also evident that more therapy is of benefit to people with stroke and the aim of this RCT is to maximise motor recovery by allowing the patient to practice activities with his/her family outside of the routine treatment hours. The qualitative data will allow the researchers a deeper insight into the impact of the programme of the patient and his/her family. Only by establishing evidence-based interventions, such as the one outlined in this protocol, can we strive to reduce the impact of this illness on the individual, the family and society.

## Competing interests

The authors declare that they have no competing interests.

## Authors' contributions

All authors contributed to the development and writing of the protocol. All authors have been involved in the drafting and revision of this manuscript and have given approval of the final manuscript.

## Pre-publication history

The pre-publication history for this paper can be accessed here:


